# New earthworm species of the genus *Amynthas* Kinberg, 1867 from Thailand (Clitellata, Oligochaeta, Megascolecidae)

**DOI:** 10.3897/zookeys.90.1121

**Published:** 2011-04-14

**Authors:** Ueangfa Bantaowong, Ratmanee Chanabun, Piyoros Tongkerd, Chirasak Sutcharit, Samuel W. James, Somsak Panha

**Affiliations:** 1 Animal Systematics Research Unit, Department of Biology, Faculty of Science, Chulalongkorn University, 254 Phayathai Road, Pathumwan, Bangkok 10330, Thailand; 2 Biodiversity Institute, University of Kansas, Lawrence, KS 66045, USA

**Keywords:** *Amynthas*, Earthworm, Taxonomy, New species, Thailand

## Abstract

Four new species of terrestrial earthworms from the *zebrus*-group in the genus *Amynthas* Kinberg, 1867, are described from Nan province, north Thailand: *Amynthas phatubensis* **sp. n.**, from Tham Pha Tub Arboretum, *Amynthas tontong* **sp. n.**, from Tontong Waterfall, *Amynthas borealis* **sp. n.**, from Chaloemprakiat district, and *Amynthas srinan* **sp. n.**, from Srinan National Park.After comparing with the two closely related Laos species *Amynthas chandyi* Hong, 2008 and *Amynthas namphouinensis* Hong, 2008, the four new species show clear morphological differences, and also it is confirmed that there are no previous records of the species described here. *Amynthas phatubensis* **sp. n.** is the largest (longest) sized of these earthworms and is the only species that lives in limestone habitats. The genital characters are different among them and also from the two Laotian species. Molecular systematics would be a good method for further analysis of the diversity and species boundaries in SE Asian *Amynthas*.

## Introduction

**Figure 1.**
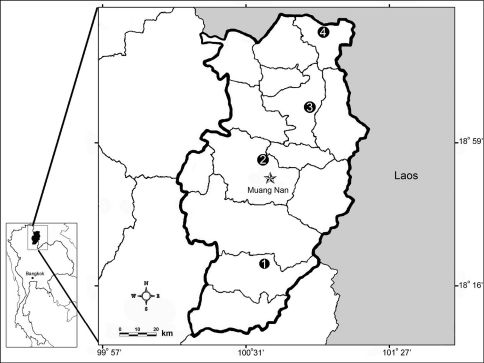
Map of type locality of **1** *Amynthas srinan* sp. n. from Srinan National Park, Nan province, **2** *Amynthas phatubensis* sp. n. from Tham Pha Tub Arboretum, Nan province, **3** *Amynthas tontong* sp. n. from Tontong Waterfall, Pua district, Nan province and **4** *Amynthas borealis* sp. n. from a small hill near Chaloemprakiat district, Nan province.

Previous taxonomic publications on, or including, the Megascolecidae (*sensu* Blakemore 2000) of Thailand are comprised of those of Gates (1972), Sims and Easton (1972) and Blakemore (2006b, 2008, 2011) and Blakemore et al. (2007). Collectively, in these publications, 32 species are recorded for Thailand, belonging to five genera (*Amynthas* Kinberg, 1867, *Lampito* Kinberg, 1867, *Metaphire* Sims & Easton, 1972, *Polypheretima* Michaelsen, 1934 and *Perionyx* Perrier, 1872). The genus *Amynthas* is one of the dominant terrestrial earthworm genera that occurs throughout Thailand and nearby countries. From the classifications by Sims and Easton (1972) and reports by Blakemore (2006b, 2011) and Somniyam (2008), it would seem that 14 species from this genus have been recorded from many areas in Thailand (Table 1). However, in addition Kosavititkul (2005) has reported six species of *Amynthas* from Khao Yai National Park, which included three unknown species, Chantaravisoot (2007) reported five species of *Amynthas* from various areas in Thailand that were all commented to be new to science, and Somniyam (2008) recorded seven *Amynthas* species from Nakhonratchasima province of which many are still unidentified. Outside of Thailand, recent publications have included that by James (2004) who described a new species (*Amynthas heaneyi*) from the Philippines; Shen and Yeo (2005) who reported four *Amynthas* species in Singapore, and Hong (2008) who described two species (*Amynthas chandyi* and *Amynthas namphouinensis*) from Laos, and also reported some publications by Vietnamese who studied the earthworm fauna in Laos and described *Amynthas xuongmontis*. From the above data it is clear that there are still many species waiting to be discovered and described. The Animal Systematics Research Unit, Chulalongkorn University’s members have surveyed terrestrial earthworms throughout Thailand since 2005 and a part of their results has been summarized in Chantaravisoot (2007). In the present paper we describe an additional four new species belonging to the *zebrus*-group, a provisional assemblage designated by Sims and Easton (1972). Each of these new species is known only from its type locality, but as more intensive collecting is undertaken in Thailand and other Asian countries, the known range and habitats of these species may be extended. The habitats of all four new species were in the topsoil layer covered with leaf litter of deciduous forests. The localities were in Nan province, in the north of Thailand, as shown in Figure 1.

**Table 1.** Morphological characteristics comparison of *Amynthas* species recorded in Thailand. The morphological characters are from the original description of each nominal species, except for the character with (*) are from Gates (1972). (**) indicate the known localities of *Amynthas* species in Thailand taken from Gates (1972), Kosavititkul (2005) and Somniyam (2008). Species group are as per Sims and Easton (1972)

Species	Species group	Body length(mm)	Number of segments	Sperma-thecal pores	Genital markings	Genital marking glands	Seminal vesicles	Prostate glands	Intestinal caeca	Distribution**
*Amynthas hupbonensis* (Stephenson, 1931)	aeruginosus	225	142	7/8–8/9	absent	absent	large in XI, XII	XVI–XX	manicate,XXVII–	Chonburi
*Amynthas alexandri* (Beddard, 1900)	corticis	145	133	5/6–8/9	absent	absent	XI, XII	XVII–XX	simple,XXVII–XX	Chiengrai, Chiengmai, Nakornratchasima, Bangkok, Chonburi
*Amynthas comptus* (Gates, 1932)	corticis	197–260*	120–134*	5/6–8/9	three trios on 18/19–20/21	sessile	larger in XI, XII	XVIII	simple,XXVII–XXIII	Phrae
*Amynthas exiguus austrinus* (Gates, 1932)	corticis	33–68	73–102	5/6–8/9	two pairs on 17/18,18/19	absent	small in XI, XII	XVII–XX	simple,XXVII–XXIV	Chiengmai
*Amynthas exiguus exiguus* (Gates, 1930)	corticis	43	90	5/6–8/9	paired on vii, viii, xix, xx	absent	small in XI, XII	XVII–XIX	simple,XXVII–XXIV	Phrae
*Amynthas longicauliculatus* (Gates, 1931)	corticis	170	138	5/6–8/9	three pairs on 18/19– 20/21	sessile	XI, XII	XVIII	simple,xxvii-xxiv	Chiengmai, Lumphun, Nakornratchasima
*Amynthas manicata decorosa* (Gates, 1932)	corticis	40	60	5/6–8/9	one pair on xviii	sessile	large in XI, XII	XVII–XIX	manicate,XXVII–XXII	Chiengmai
*Amynthas mekongianus* (Cognetti, 1922)	corticis	1 meter	370	5/6-8/9	absent	absent	10/11-11/12	XVII-XVIII	simple,XXVII-XXIII	Chiengrai
*Amynthas defecta* (Gates, 1930)	gracilis	>78	>49	5/6–7/8	absent	absent	small in XI, XII	absent	manicate,XXVII–XXVI	Nakornratchasima
*Amynthas gracilis* (Rosa, 1891)	gracilis	100	88–95	5/6–7/8	clusters on xviii	stalked*	XI, XII*	XVII–XXIII	simple,XXVII–XXIV*	Dor Kiu Koh Ma,north Thailand
*Amynthas papulosus* (Rosa, 1896)	gracilis	45–50	110–115	5/6–7/8	transverse row on XVII–XIX	stalked*	XI, XII	XVI–XXI	simple,XXVII–XXII*	Yala
*Amynthas morrisi* (Beddard, 1892)	morrisi	52	93	5/6–6/7	near spermathecal pore	stalked	XI, XII*	XVII–XXIII*	simple,XXVII–XXIV*	Chiengmai
*Amynthas fucosus* (Gates, 1933)	sieboldi	120	114	6/7–8/9	two pairs on 17/18, 18/19	sessile	large in XI, XII	XVII–XX	simple,XXVII–XVII	Nakornratchasima
*Amynthas siam* Blakemore, 2011	sieboldi	>70	>73	6/7-8/9	one pair postsetal on XVIII	sessile	XI, XII	XVIII-	simple,XXVII-	Sakon Nakhon

Since none of the four species described in this paper seems to fit the descriptions of species described in the past, the purpose of this paper is to formally describe these species as new to science. Their descriptions follow.

## Material and methods

Earthworms were collected from deciduous forests in many areas in Nan province, north of Thailand, by carefully digging up the topsoil near casts and by hand sorting the leaf litter. The worms were killed in 30% (v/v) ethanol, photographed, transferred to 5% (w/v) formalin for fixation for approximately 12 hours, and then transferred to 70% (v/v) ethanol for longer term preservation and subsequent morphological studies.

Duplicate specimens and/or tissue samples (in the cases of morphotypes determined to be unique on field inspection) were preserved in 95% ethanol for molecular data and DNA barcoding. Tissues were sent to the Canadian Center for DNA Barcoding (Hebert et al. 2003a, b) and processed according to their standard protocols (Hajibabaei et al. 2005; Ivanova et al. 2006; Ratnasingham and Hebert 2007). DNA barcode data are provided for paratype specimens of the first two species described in this paper. The sequences were aligned with Clustal X using default settings, and the resulting Neighbor-Joining tree (Saitou and Nei 1987) was used to identify barcode clusters. These clusters were matched to OTUs identified from quick examination of external characters. Inter- and intra- cluster genetic distances were calculated in MEGA 4 (Tamura et al. 2007) using the Kimura two parameter distance (Kimura 1980) using gamma-distributed rates among sites, pairwise deletion of sites with missing data, and using all substitution types and codon positions.

**Figure 2.**
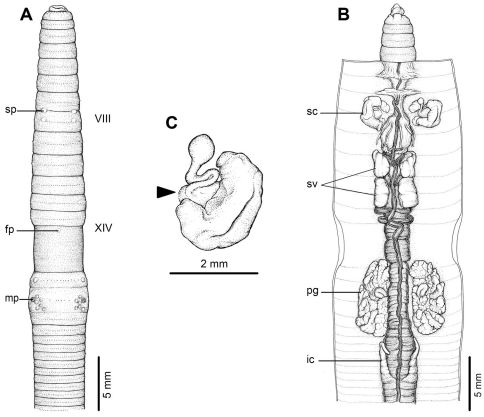
External and internal morphology of holotype (CUMZ 3204) of *Amynthas phatubensis* sp. n. **A** External ventral view, **B** internal dorsal view and **C** spermatheca, and black arrow indicates the connection of the spermatheca and spermathecal pore.

**Figure 3.**
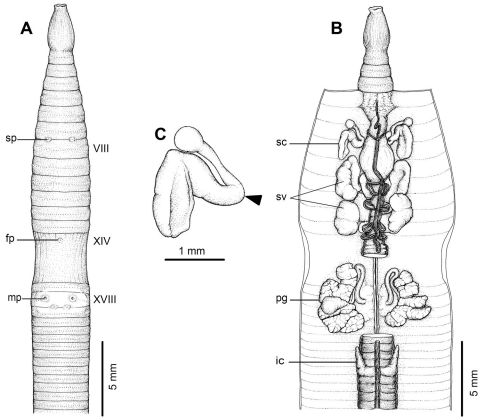
External and internal morphology of holotype (CUMZ 3206) of *Amynthas tontong* sp. n. **A** External ventral view, **B** internal dorsal view and **C** spermatheca, and black arrow indicates the connection of the spermatheca and spermathecal pore.

**Figure 4.**
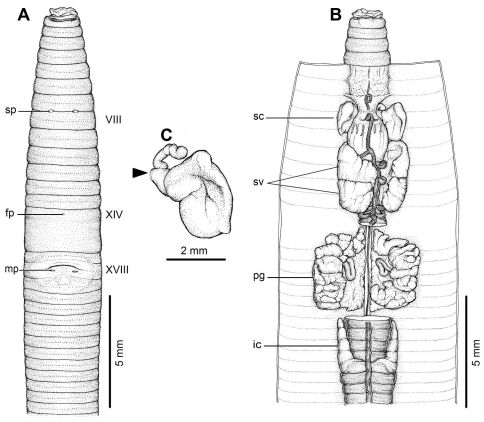
External and internal morphology of holotype (CUMZ 3208) of *Amynthas borealis* sp. n. **A** External ventral view, **B** internal dorsal view and **C** spermatheca, and black arrow indicates the connection of the spermatheca and spermathecal pore.

**Figure 5.**
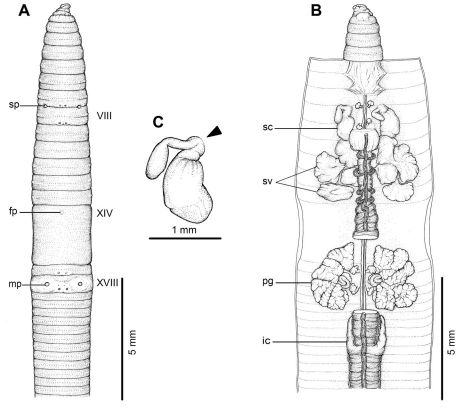
External and internal morphology of holotype (CUMZ 3210) of *Amynthas srinan* sp. n. **A** External ventral view, **B** internal dorsal view and **C** spermatheca, and black arrow indicates the connection of the spermatheca and spermathecal pore.

The descriptions of each species were made during observation under a Stemi DV 4 ZEISS stereoscopic light microscope. Drawings were made of the body segments and the distinct external characters and internal organs, as mentioned above, and are shown in Figures 2–5 for the four new species, respectively. The number of segments and the body width and length were measured in both full adults and juveniles, and are presented as the range (min-max) and mean±one standard deviation.

Type specimens housed at the Department of Biology, Faculty of Science, National University of Laos, Vientiane, Laos (BDNUL), of the two closely related Laos species, *Amynthas chandyi* Hong, 2008 and *Amynthas namphouinensis* Hong, 2008, have been critically studied and compared with the new species of this report.

Holotype and paratype specimens have been deposited in the Chulalongkorn University, Museum of Zoology, Bangkok, Thailand (CUMZ). Additional paratypes are housed in the Biozentrum Grindel und Zoologisches Museum, Hamburg, Germany (UHH), and the Natural History Museum, London (NHM).

Anatomical abbreviations: fp, female pore; ic, intestinal caeca; mp, male pores; pg, prostate gland; sc, spermathecae; sp, spermathecal pores; sv, seminal vesicles.

## Systematics

**Genus *Amynthas* Kinberg, 1867**

Type species. *Amynthas aeruginosus* Kinberg, 1867, by monotypy.

### Amynthas phatubensis

Panha & Bantaowong sp. n.

urn:lsid:zoobank.org:act:299379EB-C7CE-4B89-8A40-40E3122DCAB9

http://species-id.net/wiki/Amynthas_phatubensis

Figs 1 2 

#### Description of holotype:

Dimensions; 110 mm by 4.3 mm at segment X, 4.3 at segment XX, 4.0 mm at clitellum; body cylindrical with 108 segments. Setae regularly distributed around segmental equators, numbering 51 at VII, 60 at XX, 15 between mp, setae formula AA:AB:ZZ:ZY= 1:1:1:1 at XIII with no ventral gaps. Single fp at XIV. Prostomium epilobic with tongue open. First dorsal pore at 5/6. Clitellum annular XIV–XVI with no setae.

A pair of mp is located ventro-laterally in XVIII, or at 9th seta line, 0.33 circumference apart ventrally, convex structure; distance between mp 4.2 mm. Porophores (protuberances bearing male aperture), papilla-like structures. Each mp surrounded by six flat, circular genital markings almost the same diameter as mp, also one pair is equatorial in XVII in line with the male pores. One pair of sp in intersegmental furrow 7/8, distance between pores 0.32 circumference ventrally apart; distance between sp 3.5 mm. Genital markings, rounded, flat, located close to sp, postsetal paired on VII very near 7/8, presetal paired on VIII.

Septa 5/6 and 6/7 thick, 7/8 thin, 8/9 and 9/10 absent, 10/11–13/14 thin. Gizzard large within VIII–X, intestinal origin in XV, no lymph glands observed. Typhlosole small from XXVII. Intestinal caeca originate from XXVII extending forward to XXIII, simple, long finger-shape. Hearts esophageal in X–XIII. Holandric; testes and funnels in ventrally joined sacs in X–XI. Seminal vesicles paired in XI–XII. Prostates in XVII–XX; prostatic ducts U-shape. Genital marking glands absent.

Ovaries in XIII. Sc one pair in VIII; ampulla large ovate sac, duct stout, short; long stalked diverticulum, convoluted kinks enclosed within membrane, spherical knob terminal. No nephridia on spermathecal ducts. A large sessile genital marking gland corresponding to each external genital marking in VII–VIII.

All the key morphological characters of the holotype and paratype specimens are given in Table 2.

**Table 2.** Holotype and Paratype dimension and other morphological characteristics of *Amynthas phatubensis* Panha & Bantaowong, sp. n.

CharactersTypes	Body length (mm)	Number of segments	Location of genital markings		First dorsal pore	Number of setae			Prostate glands	Intestinal caeca
			Preclitellum	Postclitellum		VII	XX	Between male pore		
Holotype CUMZ 3204	110	108	VII, VIII	XVII, XVIII	5/6	51	60	15	XVII–XX	XXVII–XXIII
Paratype CUMZ 3205										
1	90	96	VII, VIII	XVIII	5/6	60	58	15	XVII–XXI	XXVII–XXIV
2	105	107	VII, VIII, IX	XVII, XVIII, XIX	5/6	52	58	12	XVII–XX	XXVII–XXIV
3	100	105	VII, VIII, IX	XVIII	5/6	53	60	9	XVII–XX	XXVII–XXIV
4	80	86	VII, VIII	XVII, XVIII, XIX	5/6	53	65	13	XVII–XX	XXVII–XXIV
5	120	96	VII, VIII	XVIII	5/6	58	68	11	XVII–XX	XXVII–XXIV
6	101	85	VII, VIII	XVIII	5/6	51	59	9	XVII–XX	XXVII–XXIV
7	131	86	VII, VIII	XVII, XVIII	5/6	64	67	15	XVII–XXI	XXVII–XXII
8	108	98	VII, VIII	XVII, XVIII	5/6	58	62	15	XVII–XXI	XXVII–XXII
9	116	99	VII, VIII	XVII, XVIII	5/6	53	64	11	XVII–XXI	XXVII–XXIII
10	89	92	VII, VIII	XVII, XVIII	5/6	64	58	12	XVII–XX	XXVII–XXIV
11	99	106	VII, VIII, IX	XVII, XVIII	5/6	60	63	13	XVII–XXI	XXVII–XXIV
12	112	112	VII, VIII	XVII, XVIII	5/6	52	58	11	XVII–XX	XXVII–XXIII
13	142	110	VII, VIII	XVII, XVIII	5/6	49	58	7	XVII–XX	XXVII–XXIV
14	137	108	VII, VIII, IX	XVII, XVIII	5/6	62	65	11	XVII–XX	XXVII–XXIII
15	80	85	VII, VIII, IX	XVII, XVIII	5/6	54	60	13	XVII–XX	XXVII–XXIV
16	89	111	VII, VIII, IX	XVII, XVIII	5/6	57	59	14	XVII–XXI	XXVII–XXIII
17	84	105	VII, VIII	XVII, XVIII	5/6	52	59	11	XVII–XX	XXVII–XXIV
18	148	112	VII, VIII	XVII, XVIII	5/6	51	58	12	XVII–XX	XXVII–XXII
19	109	114	VII, VIII	XVII, XVIII	5/6	64	59	12	XVII–XX	XXVII–XXII
20	144	107	VII, VIII	XVII, XVIII	5/6	53	60	11	XVII–XXI	XXVII–XXIV
21	84	108	VII, VIII	XVII, XVIII	5/6	64	61	15	XVII–XX	XXVII–XXII

#### Variation:

The holotype measures 110 mm body length with 108 segments; the twenty one paratypes range in size from 80–148 mm (108±21.93 mm) body length with 85–114 segments (Table 2).

#### Type locality:

Tham Pha Tub Arboretum, Nan province, Thailand, 18°51'16.4"N, 100°44'10.1"E, 265 meters elevation (11th October 2009). We also collected another lot of further specimens from Tontong Waterfall, Nan province (location 3 in Figure 1), which is located about a hundred kilometers north of the type locality.

#### Etymology:

This species was named after the type locality, Tham Pha Tub Arboretum.

#### Type material:

The holotype (CUMZ 3204) and 15 paratypes (CUMZ 3205) and 10 paratypes (CUMZ 3212) are deposited in Chulalongkorn University, Museum of Zoology. Another four paratypes will be deposited in the Biozentrum Grindel und Zoologisches Museum, Hamburg, Germany (UHH), and three paratypes in the Natural History Museum, London (NHM).

#### Habitat:

Found in the top soil at about 10 cm depth, the soil surface was covered with leaf litter in a deciduous limestone forest at Tham Pha Tub Arboretum. The soil was carefully dug close to the casts. Many ariophantid snails, *Cryptozona siamensis* Pfeiffer, 1856 were on the ground or under leaf litter.

#### Diagnosis:

*Amynthas phatubensis* sp. n. is a medium to large sized terrestrial earthworm with a pair of mp surrounded by six genital papillae on segment XVIII. Within the *zebrus*-group, this species is diagnosed by the unique combination of dorsal pores in 5/6, simple digitate caeca, ventrally joined testis sacs, genital marking glands in the spermathecal segments, and the spermathecal characters of the large ovate ampulla, stalked diverticulum whose folds are membrane-bound, and spherical knob terminal diverticulum sac.

#### Remarks:

**Figure 6.**
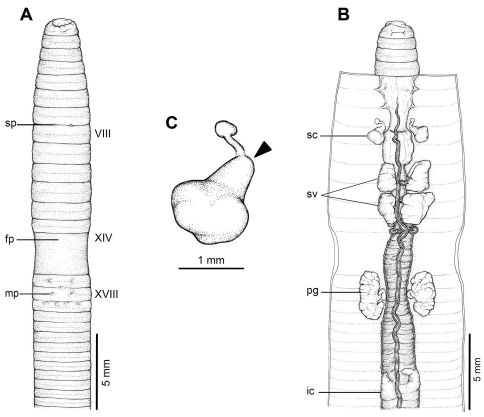
External and internal morphology of holotype (BDNUL 0001) of *Amynthas namphouinensis* Hong, 2008 **A** External ventral view, **B** internal dorsal view and **C** spermatheca, and black arrow indicates the connection of the spermatheca and spermathecal pore.

**Figure 7.**
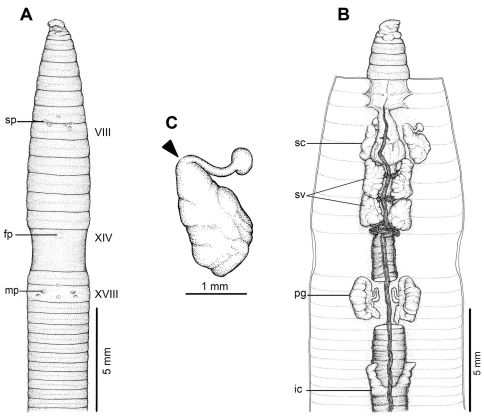
External and internal morphology of holotype (BDNUL 0002) of *Amynthas chandyi* Hong, 2008 **A** External ventral view, **B** internal dorsal view and **C** spermatheca, and black arrow indicates the connection of the spermatheca and spermathecal pore.

*Amynthas phatubensis* sp. n. has very simple characteristics of the genus, but among these, only the superficial male pores are external. In most newly collected specimens it was difficult to observe the pores or marks on the bodies. However, after preservation they can be seen more clearly. The internal organs are much more easily discerned. This new species is quite distinct when compared to the two closely related species from Laos, *Amynthas chandyi* Hong, 2008 and *Amynthas namphouinensis* Hong, 2008, which belong in the same *zebrus*-group. The two Laos species are a little bit smaller than *Amynthas phatubensis* sp. n., especially *Amynthas chandyi*. Even though *Amynthas namphouinensis* is much closer in appearance to *Amynthas phatubensis* sp. n., there are distinct differences between the type specimens (Figs 6 and 7). For example, the distance between the mp of *Amynthas phatubensis* sp. n. is 4.2 mm for the holotype and range from 3.0–4.5 mm (4.27±0.57mm), while for *Amynthas namphouinensis* this was significantly smaller, ranging from 1.4–1.5 mm. The distance between a pair of sp is also different, being 3.5–4.5 mm (4.12±0.4 mm) for *Amynthas phatubensis* sp. n. and 1.4–2.0 mm in *Amynthas namphouinensis*. The distance between the male pores as a fraction of the estimated circumference of the 18th segment is 0.30–0.33 in *Amynthas phatubensis* sp. n., but 0.10–0.14 circumference apart in *Amynthas namphouinensis*. Moreover, *Amynthas phatubensis* sp. n. has no genital marking glands on segments XVII–XIX, where *Amynthas namphouinensis* has sessile genital marking glands, but contains two distinct genital marking glands located close to sc that are absent in *Amynthas namphouinensis*.

Two populations of *Amynthas phatubensis* sp. n. were sampled, one from the type locality and one from Tontong waterfall. Distinct DNA barcode clusters corresponding to these populations had intra-cluster Kimura 2 parameter distances of 0.023 (N=9) and 0.016 (N=5) respectively. The inter-cluster divergence between the two populations is 0.084. Based on the morphological unity and the fact that the divergence is less than that usually seen between congeneric species pairs of earthworms (Chang et al. 2007; Pérez-Losada et al. 2005, James et al. 2010), we choose to maintain the two populations as representing one species. By contrast, the inter-cluster divergence between these populations and three other morpho-species with the same spermathecal battery, from the same two sites is in the range of 0.269-0.294. A consensus sequence from the type locality specimens is in Appendix 1. Another use of COI barcode sequence from type material is in Blakemore et al. (2010).

### Amynthas tontong

Panha & Bantaowong sp. n.

urn:lsid:zoobank.org:act:3317146B-143D-4EFC-A0C9-60262073BAFF

http://species-id.net/wiki/Amynthas_tontong

Figs 1 3 

#### Description of Holotype:

Dimensions; 53 mm by 2.7 mm at segment X, 2.6 at segment XX, 2.2 mm at clitellum; body cylindrical with 80 segments. Setae regularly distributed around segmental equators, numbering 42 at VII, 52 at XX, no visible setae between mp, setae formula AA:AB:ZZ:ZY= 1.5:1:1:1 at XIII. Single fp at XIV. Prostomium epilobic. First dorsal pore at 5/6. Clitellum annular XIV–XVI with no setae.

A pair of indistinct rounded mp in XVIII, 0.19 mm circumference apart ventrally; distance between mp 1.0 mm at 5th seta line. Genital markings closely paired located medial to male pore level in intersegment 18/19. Sp paired in 7/8 at 4th seta line, each small, lip-like structure within porophore, 0.10 circumference apart ventrally; distance between sp 1.0 mm. Genital markings near sp absent.

Septa 5/6 and 6/7 thick, 7/8 thin, 8/9 and 9/10 absent 10/11–13/14 thin. Gizzard large within VIII–X, intestinal origin in XV, no lymph glands observed. Typhlosole small from XXVII. Ic originated from XXVII extending forward to XXV, simple finger-shape. Hearts esophageal in X–XIII. Holandric; testes and funnels in ventrally joined sacs in X–XI. Sv paired in XI–XII. Prostates in XVIII; prostatic ducts long slender with U-shape. Genital marking glands absent.

Ovaries in XIII. Sc one pair in VIII; ampulla thumb shape, duct stout, shorter than ampulla. Diverticulum slender stalk with spherical knob terminal, no genital marking glands observed.

All the key morphological characters of the holotype and paratype specimens are given in Table 3.

**Table 3.** Holotype and Paratype dimension and other morphological characteristics of *Amynthas tontong* Panha & Bantaowong sp. n.

CharactersTypes	Body length (mm)	Number of segments	Genital markings	First dorsal pore	Number of setae		Between male pore	Prostate glands	Intestinal caeca
					VII	XX			
HolotypeCUMZ 3206	53	80	XVIII	5/6	42	52	0	XVII–XX	XXVII–XXIV
ParatypeCUMZ 3207									
1	41	71	XVIII	5/6	41	53	0	XVI–XVIII	XXVII–XXV
2	39	74	XVIII	5/6	42	52	0	XVII–XX	XXVII–XXIV
3	41	73	XVIII	5/6	46	55	0	XVII–XIX	XXVII–XXIII

#### Variation:

The holotype measures 53 mm body length with 80 segments; the three paratypes range in size from 39–41 mm (40.33±1.15 mm) body length with 71–74 segments (Table 3).

#### Type locality:

Tontong Waterfall, Nan province, Thailand, 19°12'35.9"N, 101°04'13.7"E, 1,128 meters elevation (10th October 2009).

#### Etymology:

This species was named after the type locality, Tontong Waterfall.

#### Type material:

The holotype (CUMZ 3206) and two paratypes (CUMZ 3207) are deposited in Chulalongkorn University, Museum of Zoology. Another paratype will be deposited in the Biozentrum Grindel und Zoologisches Museum, Hamburg, Germany (UHH).

#### Habitat:

Found in the top soil at about 10 cm depth, the soil surface covered with leaf litter of deciduous forest which originated at the Tontong Waterfall area. The soil was carefully dug close to surface casts. Most surrounding areas have been modified to agricultural fields.

#### Diagnosis:

*Amynthas tontong* sp. n. is a small sized terrestrial earthworm with a close indistinct pair of male pores with a pair of genital markings in intersegment 18/19. Spermathecae consists of a thumb shaped ampulla and a spherical terminal knob shaped diverticulum. Genital marking glands absent, first dorsal pore in 5/6, intestinal caeca simple, intestinal origin XV, septa 8/9/10 absent, testis sacs joined ventrally.

#### Remarks:

*Amynthas tontong* sp. n., along with *Amynthas srinan* sp. n. and *Amynthas exiguus exiguus*, is one of the smallest sized *Amynthas* ever recorded in Thailand. The basic external characters are easily seen in both newly collected and preserved materials. Compared with the two other closely related species from Laos, *Amynthas chandyi* Hong, 2008 and *Amynthas namphouinensis* Hong, 2008, which belong in the same *zebrus*-group, *Amynthas chandyi* is similar to *Amynthas tontong* sp. n. However, it differs in the specific details of the significant characters, such as the distance between the mp in *Amynthas tontong* sp. n. is 1.0 mm for the holotype and ranged from 1.0–1.2 mm (0.93±0.12 mm), while in *Amynthas chandyi* it ranged from 1.5–2.4 mm. The distance between the male pores as a fraction of the estimated circumference of the 18th segment is 0.15–0.19 in *Amynthas tontong* sp. n., but 0.14–0.32 in *Amynthas chandyi*. The arrangement of the genital markings of both species are totally different, and the distance between a pair of sp is also different, being 0.8–1.0 mm (1.1±0.1 mm) in *Amynthas tontong* sp. n. and 1.2–1.5 mm for *Amynthas chandyi*. Moreover, *Amynthas tontong* sp. n. has no genital markings near to the sp,whilst *Amynthas chandyi* exhibits circular genital markings in various locations, paired or single mid ventral in VII, VIII; usually 3 or 4 in total.

Alcohol-preserved paratype specimens of *Amynthas tontong* sp. n. belonged to a single DNA barcode cluster, with an intra-cluster divergence of 0.005 (N=3), and diverging from *Amynthas phatubensis* sp. n. by 0.294, and by 0.189 for an undescribed species. An undescribed morph at Tham Pha Tub diverged by 0.100, and may represent a subspecies. A consensus sequence is in Appendix 1.

### Amynthas borealis

Panha & Bantaowong sp. n.

urn:lsid:zoobank.org:act:C2BE17F8-A721-4736-9809-EF9ABDAB0C03

http://species-id.net/wiki/Amynthas_borealis

Figs 1 4 

#### Description of Holotype:

Dimensions; 54 mm by 3.5 mm at segment X, 3.8 at segment XX, 3.5 mm at clitellum; body cylindrical with 89 segments. Setae regularly distributed around segmental equators, numbering 39 at VII, 51 at XX, no visible setae between mp, setae formula AA:AB:ZZ:ZY= 2:1:1.5:1 at XIII. Single fp at XIV. Prostomium epilobic. First dorsal pore at 5/6. Clitellum annular XIV–XVI with no setae.

Mp pocket-like structures indistinctly occur in XVIII, 0.10 circumference apart ventrally; distance between mp 1.0 mm; porophores small, lip-like and surrounded by an elevated skin fold at medial pores, and there is a long ridge with a sharp posterior boundary traversing the body in front of the mp. Genital markings absent. Sp paired in 7/8 at 4th seta line, 0.10 circumference apart ventral; distance between sp 1.0 mm. Genital markings absent.

Septa 5/6 and 6/7 thick, 7/8 thin, 8/9 and 9/10 absent, 10/11–13/14 thin. Gizzard large within VIII–X, intestinal origin in XV, no lymph glands observed. Typhlosole small from XXVII. Ic originated from XXVII extending forward to XXV, simple finger-shape. Hearts esophageal in X–XIII. Holandric; testes and funnels in ventrally joined sacs in X–XI. Sv paired in XI–XII. Prostates in XVIII; prostatic ducts long slender bent in U-shape. Genital marking glands absent.

Ovaries at XIII. Sc one pair in VIII; ampulla large sac-shape, flattened by gizzard, narrow duct shorter than ampulla. Diverticulum with elongated tubular shape, stalk attached to duct near body wall, with no genital marking glands.

All the key morphological characters of the holotype and paratype specimens are given in Table 4.

**Table 4.** Holotype and Paratype dimension and other morphological characteristics of *Amynthas borealis* Panha & Bantaowong, sp. n.

CharactersTypes	Body length (mm)	Number of segments	Genital markings	First dorsal pore	Number of setae		Between male pore	Prostate glands	Intestinal caeca
					VII	XX			
HolotypeCUMZ 3208	54	89	Absent	5/6	39	51	0	XVII–XIX	XXVII–XXV
ParatypeCUMZ 3209									
1	45	87	Absent	5/6	51	48	0	XVII–XX	XXVII–XXIV
2	42	78	Absent	5/6	49	45	0	XVIII–XIX	XXVII–XXIII
3	44	79	Absent	5/6	51	50	0	XVII–XX	XXVII–XXIII
4	42	86	Absent	5/6	54	41	0	XVIII–XIX	XXVII–XXIV
5	44	85	Absent	5/6	40	40	0	XVIII–XIX	XXVII–XXIV
6	42	85	Absent	5/6	46	48	0	XVII–XIX	XXVII–XXIV
7	42	77	Absent	5/6	44	50	0	XVII–XX	XXVII–XXV
8	42	83	Absent	5/6	48	52	0	XVII–XIX	XXVII–XXV

#### Variation:

The holotype measures 54 mm body length with 89 segments; the eight paratypes range in size from 42–45 mm (42.87±1.25 mm) body length with 77–87 segments (Table 4).

#### Type locality:

Chaloemprakiat district, Nan province, Thailand, 19°34'48.5"N, 101°04'53.1"E, 513 meters elevation (7th August 2010).

#### Etymology:

The specific epithet “*borealis*” derived from Latin word “boreal” mean “north”. This name refers to the location of type locality in the north of Thailand.

#### Type material:

The holotype (CUMZ 3208) and seven paratypes (CUMZ 3209) are deposited in Chulalongkorn University, Museum of Zoology. Another two paratypes will be deposited in the Biozentrum Grindel und Zoologisches Museum, Hamburg, Germany (UHH), and another two paratypes in the Natural History Museum, London (NHM).

#### Habitat:

Found in the top soil at about 10 cm depth, the soil surface covered with the leaf litter of a deciduous limestone forest, mostly disturbed. The soil was carefully dug close to the casts.

#### Diagnosis:

*Amynthas borealis* sp. n. is a small sized terrestrial earthworm small male pores, a transverse ridge anterior to the male pores in XVII, and no genital markings. One pair of sc in VIII, each spermathecae consists of a large sac-shaped ampulla and elongated tubular shaped diverticulum. Testis sacs joined ventrally, intestinal origin XV, intestinal caeca simple, first dorsal pore in 5/6.

#### Remarks:

*Amynthas borealis* sp. n. is one of the smaller *Amynthas*. The characteristic male field is difficult to see in newly collected specimens but can be clearly observed after preservation. Compared with the two other closely related species from Laos, *Amynthas chandyi* and *Amynthas namphouinensis*, which belong in the same *zebrus*-group, *Amynthas chandyi* is similar to *Amynthas borealis* sp. n. However, distinctive differences include the distance between mp of the new species, being 1.0 mm in the holotype with a range of 0.8–1.0 mm (0.95±0.09 mm) in *Amynthas borealis* sp. n. compared to 1.5–2.4 mm. The distance between the male pores as a fraction of the estimated circumference of the 18th segment is 0.10–0.14 in *Amynthas borealis* sp. n., but 0.14–0.32 in *Amynthas chandyi*. There are no genital markings in the new species; the distance between a pair of sp is also different, being 0.5–1.0 mm (0.9±0.19 mm) in the new species compared to 1.2–1.5 mm for *Amynthas chandyi*. Moreover, *Amynthas borealis* sp. n. has no genital marking glands at all, whilst *Amynthas chandyi* exhibits circular genital markings in various locations, paired or single mid ventral in VII and VIII; usually 3 or 4 in total.

### Amynthas srinan

Panha & Bantaowong sp. n.

urn:lsid:zoobank.org:act:C3EC91E6-B29A-4C72-908F-1858DE7F21DA

http://species-id.net/wiki/Amynthas_srinan

Figs 1 5 

#### Description of Holotype:

Dimensions; 47 mm by 1.8 mm at segment X, 2.3 at segment XX, 2.3 mm at clitellum; body cylindrical with 77 segments. Setae regularly distributed around segmental equators, numbering 36 at VII, 42 at XX, four between mp, setae formula AA:AB:ZZ:ZY= 1.5:1:2:1 at XIII. Single fp at XIV. Prostomium epilobic with tongue open. First dorsal pore at 4/5 or 5/6. Clitellum annular XIV–XVI with no setae.

Mp on circular porophores in XVIII, 0.30 circumference apart ventrally; distance between mp 1.5 mm. Genital markings small, postsetal, closely paired near mid ventral of XVII and XVIII. Sp paired in 7/8 at 6th setal lines, 0.26 circumference apart ventrally; distance between sp 1.5 mm. Genital markings tiny, closely paired on near mid ventral of VII and VIII.

Septa 5/6 and 6/7 thick, 7/8 thin, 8/9 and 9/10 absent, 10/11–13/14 thin. Gizzard globular within VIII–X, intestinal origin in XV, no lymph glands observed. Typhlosole small from XXVII. Ic originated from XXVII extending forward to XXIII, long finger-shape. Hearts esophageal in X–XIII. Holandric; testes and funnels in ventrally joined sacs in X–XI. Sv paired in XI–XII. Prostates in XVIII, extending between XVII–XX; prostatic ducts tightly folded twice. Genital marking glands paired in XVII and XVIII corresponding to external genital papillae, each consisting of a stalk with terminal multi-lobed glandular part.

Ovaries in XIII. Sc one pair in VIII; ampulla oval to kidney-shaped, with stout duct shorter than ampulla. Diverticulum with oval bulb terminal, stalk attached to duct near body wall. Genital markings stalked, corresponding to external genital papillae; each gland small consisting of a stalk with terminal multi-lobed glandular part.

All the key morphological characters of the holotype and paratype specimens are given in Table 5.

**Table 5.** Holotype and Paratype dimension and other morphological characteristics of *Amynthas srinan* Panha & Bantaowong, sp. n.

CharactersTypes	Body length (mm)	Number of segments	Location of genital markings		First dorsal pore	Number of setae		Between male pore	Prostate glands	Intestinal caeca
			preclitellum	postclitellum		VII	XX			
HolotypeCUMZ 3210	47	77	VII, VIII	XVII, XVIII	5/6	36	42	4	XVII–XX	XXVII–XXIII
ParatypeCUMZ 3211										
1	35	75	VII, VIII	XVII, XVIII	5/6	40	42	6	XVII–XX	XXVII–XXV
2	44	76	VII, VIII	XVII, XVIII	5/6	36	42	5	XVII–XX	XXVII–XXIV
3	39	65	VII, VIII	XVII, XVIII	5/6	37	46	4	XVIII–XX	XXVII–XXIV
4	44	70	VII, VIII	XVII, XVIII	5/6	36	49	5	XVII–XIX	XXVII–XXIV
5	47	78	VII, VIII	XVII, XVIII	5/6	38	45	4	XVII–XX	XXVII–XXIV
6	37	68	VII, VIII	XVII, XVIII	5/6	40	44	4	XVII–XX	XXVII–XXV
7	38	77	VII, VIII	XVII, XVIII	4/5	43	48	5	XVII–XXI	XXVII–XXIV
8	37	52	VII, VIII	XVII, XVIII	4/5	38	42	4	XVII–XXI	XXVII–XXV
9	35	57	VII, VIII	XVII, XVIII	4/5	41	44	4	XVII–XX	XXVII–XXIV
10	38	78	VII, VIII	XVII, XVIII	5/6	36	40	4	XVII–XX	XXVII–XXIV
11	42	77	VII, VIII	XVII, XVIII	4/5	42	47	4	XVII–XXI	XXVII–XXIII
12	45	77	VII, VIII	XVII, XVIII	5/6	39	45	5	XVII–XX	XXVII–XXIV
13	40	77	VII, VIII	XVII, XVIII	5/6	40	48	4	XVII–XIX	XXVII–XXV
14	39	77	VII, VIII	XVII, XVIII	5/6	39	47	4	XVII–XX	XXVII–XXIV
15	43	77	VII, VIII	XVII, XVIII	5/6	40	44	4	XVII–XX	XXVII–XXIII
16	40	75	VII, VIII	XVII, XVIII	5/6	41	49	4	XVII–XX	XXVII–XXIV
17	37	75	VII, VIII	XVII, XVIII	4/5	36	46	4	XVII–XIX	XXVII–XXIV
18	36	60	VII, VIII	XVII, XVIII	5/6	40	47	5	XVII–XX	XXVII–XXIV
19	39	75	VII, VIII	XVII, XVIII	5/6	37	44	4	XVII–XX	XXVII–XXIII
20	47	78	VII, VIII	XVII, XVIII	4/5	36	42	4	XVII–XX	XXVII–XXV
21	42	71	VII, VIII	XVII, XVIII	5/6	40	46	4	XVII–XX	XXVII–XXIV
22	35	56	VII, VIII	XVII, XVIII	4/5	41	43	4	XVII–XIX	XXVII–XXIV
23	36	69	VII, VIII	XVII, XVIII	5/6	36	45	4	XVII–XX	XXVII–XXV
24	42	73	VII, VIII	XVII, XVIII	5/6	36	46	4	XVII–XX	XXVII–XXIV
25	44	76	VII, VIII	XVII, XVIII	4/5	39	47	6	XVI–XX	XXVII–XXV
26	35	69	VII, VIII	XVII, XVIII	5/6	36	44	4	XVII–XIX	XXVII–XXIII
27	38	75	VII, VIII	XVII, XVIII	5/6	37	45	4	XVII–XX	XXVII–XXIV
28	35	78	VII, VIII	XVII, XVIII	5/6	39	44	4	XVII–XIX	XXVII–XXV

#### Variation:

The holotype measures 47 mm body length with 77 segments and the first dorsal pore located at 5/6; the twenty eight paratypes range in size between 35–47 mm (39.75±4.27 mm) body length with 52–78 segments, and first dorsal pore at 4/5 (8 samples) or 5/6 (20 samples) (Table 5).

#### Type locality:

Srinan National Park, Nan province, Thailand, 18°22'11.1"N, 100°50'23.2"E, 607 meters elevation (30th September 2010).

#### Etymology:

This species was named after the type locality Srinan National Park.

#### Type material:

The holotype (CUMZ 3210) and 25 paratypes (CUMZ 3211) are deposited in Chulalongkorn University, Museum of Zoology. Another five paratypes will be deposited in the Biozentrum Grindel und Zoologisches Museum, Hamburg, Germany (UHH), and four paratypes in the Natural History Museum, London (NHM).

#### Habitat:

Found in the top soil at about 10 cm depth, the soil surface covered with leaf litters of deciduous forest. The soil was carefully dug close to the castes.

#### Diagnosis:

*Amynthas srinan* sp. n. is the smallest *Amynthas* ever collected in Thailand. Male pores on distinct round porophores, genital markings paired near mid ventral of VII, VIII, XVII and XVIII; each with genital marking glands. Each spermathecae consists of a kidney-shaped ampulla and an oval shaped diverticulum. Testes sacs ventrally joined, intestinal origin XV, intestinal caeca simple, first dorsal pores at 4/5 or 5/6.

#### Remarks:

*Amynthas srinan* sp. n., along with *Amynthas exiguus exiguus* and *Amynthas tontong* sp. n., is one of if not the smallest *Amynthas* recorded so far. It has external characteristics which are easily seen in both newly collected and preserved materials. Compared with the two other closely related species from Laos, *Amynthas chandyi* and *Amynthas namphouinensis*, which belong in the same *zebrus*-group, *Amynthas chandyi* is very similar in appearance to *Amynthas srinan* sp. n. However, they clearly differ in certain specific details of their significant characters, such as the distance between the mp which in *Amynthas srinan* sp. n. is 1.5 mm for holotype and ranged from 1.5–2.0 mm (1.41±4.27 mm), while in *Amynthas chandyi* this ranged from 1.5–2.4 mm. The distance between the male pores as a fraction of the estimated circumference of the 18th segment is 0.24–0.30 in *Amynthas srinan* sp. n., and 0.14–0.32 in *Amynthas chandyi*. This is not convincing as a diagnostic difference, because there is significant overlap with the highly variable *Amynthas chandyi*. In addition, although genital markings are clearly observed in both *Amynthas chandyi* and *Amynthas srinan* sp. n. on the sc and mp areas, *Amynthas srinan* sp. n. has a much larger number and different arrangement of such markings. The distance between pairs of sp is quite similar, being 1.5–2.0 mm (1.34±2.31mm) in *Amynthas srinan* sp. n. and 1.2–1.5 mm in *Amynthas chandyi*.

## Discussion

The genus *Amynthas* is widely distributed in the Asian continent, where it is one of the dominant genera. In Thailand it occurs in various types of lowland forest habitats, dry evergreen, moist evergreen, deciduous and limestone forests, encompassing diverse soil pH values, from acidic to alkali soils (Chantaravisoot 2007) and from clay to muddy sand substrates (Kosavititkul, 2005; Somniyam, 2008; Blakemore et al., 2007). The current four new species described here were all are found in one area (Nan province) but the four habitat types were quite diverse all the same. *Amynthas phatubensis* sp. n. was found in a limestone area with a mild alkali substrate (pH 7.5–8) of a clay loam structure, whilst the other three species were found in harder sandy clay substrates. The four new species are broadly similar (and so potentially related) to the two species described from Laos, *Amynthas chandyi* and *Amynthas namphouinensis*, but differ in both the external and internal morphological characteristics. The geographic structures of Luang Prabang Mountain and Phi Pan Nam Mountain ranges are important barriers for species from both the Thai (Nan province) and Laos side (Xayabouli province) and may have played an important part in their speciation. In addition, the Laos species live at a higher altitude than the current new described species from Thailand, and such selective adaptations may facilitate their morphological discrimination.

The four new species range in size, with respect to other *Amynthas* members, from moderate to very small, of which *Amynthas phatubensis* sp. n. is the longest. The other three species are almost the same size and close to the two Laotian species, as shown in Table 6. However, the spermathecae (sc) and genital marking locations of the four new species are clearly different from the two closely related Laos species. The four new *Amynthas* species described here belong to the *zebrus*-group, as defined by Sims and Easton (1972),in which the spermathecal pores are located on segment 7/8. The size of these four species, relative to other *Amynthas* species, varied from small to medium, ranging from 35 to 148 mm in body length and having from 52 to 114 segments. The first dorsal pore in three of the four species described here, and most of the samples of the fourth species (*Amynthas srinan* sp. n.), is located on intersegmental furrow 5/6, but with some samples of *Amynthas srinan* sp. n. showing the first dorsal pore at 4/5.

**Table 6.** Morphological characteristics for between these four new species and two know species from Laos

Characters	*Amynthas phatubensis* sp. n.	*Amynthas tontong* sp. n.	*Amynthas borealis* sp. n.	*Amynthas srinan* sp. n.	*Amynthas namphouinensis*	*Amynthas chandyi*
Body length (mm)	80–148	39–53	42–54	35–47	63–92	29–58
Number of segments	85–112	71–80	78–89	56–77	92–94	48–52
First dorsal pore	5/6	5/6	5/6	4/5, 5/6	4/5, 5/6, 6/7	5/6
Setae number						
VII	51–64	41–46	39–54	36–45	52–61	44–54
XX	58–68	52–55	40–52	42–49	53–58	44–57
between male pores	9–15	0	0	4–6	0–7	0–7
Preclitellar genital markings						
VII	2	0	0	2	0	1–2
VIII	1–7	0	0	2	0	1–2
IX	0–1	0	0	0	0	0
Postclitellar genital markings						
XVII	0–2	0	0	2	2	1
XVIII	6–12	2	0	2	0	3
XIX	0–1	0	0	0	4	1
XX	0–1	0	0	0	0	1
Prostate glands	XVII–XX	XVII–XX	XVII–XX	XVII–XX	XVII–XIX	XVI–XXI
Genital marking glands	sessile at VII, VIII	Absent	absent	stalked	sessile at XVII–XIX	absent
Intestinal caeca	simple, XXVII–XXIII	simple, XXVII–XXV	simple, XXVII–XXV	simple, XXVII–XXII	simple, XXVII–XXIV	simple, XXVII–XXIV

*Amynthas phatubensis* sp. n. is the only species that lives in limestone habitats in leaf litter and also in shallow mild alkali topsoil. The soil humidity can be quite low and is of a clay loam structure. The other three species are smaller in size and were found in almost harder, muddy sandy clay substrates. *Amynthas tontong* sp. n. lives in deeper soil of a high humidity around waterfalls. *Amynthas borealis* sp. n. and *Amynthas srinan* sp. n. are found in deciduous forests, which have mostly been modified as agricultural fields. The soil is drier and harder. The genital marking glands of *Amynthas phatubensis* sp. n. and *Amynthas srinan* sp. n. are distinct from other two species (Table 6 and Figs 2–5), whilst *Amynthas tontong* sp. n. show two postclitellar genital markings that are absent in *Amynthas borealis* (Figs 3 and 4) The diagnostic differences are shown in the dichotomous key to the sixteen Thai and two Laotian *Amynthas* species, below.

The *zebrus*-group is composed of eleven nominal species: *Metaphire hilgendorfi* (Michaelsen, 1892), *Amynthas palmosus* (Chen, 1946), *Amynthas magnipapillatus* (Qui and Wang, 1992), *Amynthas zebrus* (Benham, 1896), *Amynthas culminus* Michaelsen, 1899, *Amynthas principalis* (Michaelsen, 1932), *Amynthas xuongmontis* (Thai & Samphon, 1990), *Amynthas fasciculus* (Qui, Wang & Wang, 1993), *Amynthas heaneyi* James, 2004, *Amynthas namphouinensis* Hong, 2008 and *Amynthas chandyi* Hong, 2008. Within the *zebrus*-group, the first three species show manicate intestinal caeca, while the current newly described four species have simple finger-shaped intestinal caeca. The three latter nominal species are longer in body length (200–300 mm) compared with the size of these four new species which ranged from 35–148 mm. *Amynthas heaneyi* can be distinguished by its proandric character (James, 2004), while the four new described species are holandric. *Amynthas fasciculus* has coiled and kinked spermathecae, whereas *Amynthas phatubensis* sp. n. has large ovate ampulla, *Amynthas tontong* sp. n. has thumb shaped ampulla, *Amynthas borealis* sp. n. has sac-shape ampulla, and *Amynthas srinan* sp. n. has oval to kidney-shaped ampulla. *Amynthas xuongmontis* clearly differs from these four new species in the genital marking located on XVIII, whereas located on VII, VIII, XVII, XVIII in *Amynthas phatubensis* sp. n., located between 18/19 in *Amynthas tontong* sp. n., absent in *Amynthas borealis* sp. n. and located on VII, VIII, XVII, XVIII in *Amynthas srinan* sp. n.

### Key to Thai and two Laos species of *Amynthas*

**Table:** 

1	First spermathecal pores at 5/6	2
–	First spermathecal pores after 5/6	12
2	Two pairs of spermathecal pores	*Amynthas morrisi*
–	More than two pairs of spermathecal pores	3
3	Three pairs of spermathecal pores	4
–	More than three pairs of spermathecal pores	6
4	Genital markings absent	*Amynthas defecta*
–	Genital markings present	5
5	Genital markings clustered on XVIII	*Amynthas gracilis*
–	Genital markings transverse rows on XVII, XVIII, XIX	*Amynthas papulosus*
6	Genital markings absent	7
–	Genital markings present	8
7	Body length 1 meter or more	*Amynthas mekongianus*
–	Body length less than 300 mm	*Amynthas alexandri*
8	Genital marking glands absent	9
–	Genital marking glands present	10
9	Genital markings located on 17/18, 18/19	*Amynthas exiguus austrinus*
–	Genital markings located on VII, VIII, XIX, XX	*Amynthas exiguus exiguus*
10	Intestinal caeca, simple	11
–	Intestinal caeca, manicate	*Amynthas manicatus decorosus*
11	Genital markings, paired at 18/19, 19/20, 20/21	*Amynthas longicauliculatus*
–	Genital markings, three trios at 18/19, 19/20, 20/21	*Amynthas comptus*
12	First spermathecal pores at 6/7	13
–	First spermathecal pores after 6/7	14
13	Genital markings located on 17/18, 18/19	*Amynthas fucosus*
–	Genital marking located on XVIII	*Amynthas siam*
14	Body length more than 200 mm	*Amynthas hupbonensis*
–	Body length less than 200 mm	15
15	Genital markings absent	*Amynthas borealis* sp. n.
–	Genital markings present	16
16	Preclitellar genital markings absent	17
–	Preclitellar genital markings present	18
17	Genital marking glands absent	*Amynthas tontong* sp. n.
–	Genital marking glands present	*Amynthas namphouinensis*
18	Genital marking glands absent	*Amynthas chandyi*
–	Genital marking glands, present	19
19	Genital marking glands, sessile	*Amynthas phatubensis* sp. n.
–	Genital marking glands, stalked	*Amynthas srinan* sp. n.

## Supplementary Material

XML Treatment for Amynthas phatubensis

XML Treatment for Amynthas tontong

XML Treatment for Amynthas borealis

XML Treatment for Amynthas srinan

## Appendix 1.

DNA barcode sequences for *Amynthas phatubensis* sp. n. and *Amynthas tontong* sp. n. Positions with variable base are indicated by the appropriate ambiguity code: Y= C or T, R= A or G, K= G or T, M= A or C.

The primer sets used, LCO1490 and HCO2198, amplify a 658 bp fragment of the COI gene in a wide range of invertebrate taxa (Folmer et al 1994):

LCO1490: 5’-GGTCAACAAATCATAAAGATATTGG-3’

HCO2198: 5’-TAAACTTCAGGGTGACCAAAAAATCA-3’

Consensus of 8 sequences of *Amynthas phatubensis* sp. n. **Paratype** CUMZ 3212, GenBank Accession No. HM901031-HM901038.

AACCCTATATTTCATTTTAGGTATTTGAGCCGGTATGATTGGATCTGGAATAAGCCTACTYATCCGAATTGARTTGAGCCAACCTGGATCCTTCCTAGGCAGAGATCAGCTATACAATACCATTGTTACAGCTCATGCATTCTTAATAATYTTCTTTTTAGTTATACCCGTATTTATTGGGGGTTTTGGAAACTGATTAYKACCACTTATACTTGGRGCGCCAGATATRGCCTTTCCYCGACTAAACAACATAAGATTCTGATTGCTTCCTCCRTCRCTTATTCTATTAGTAAGCTCTGCGGCCGTRGAAAAGGGGGCYGGCACTGGATGAACTGTTTACCCMCCCCTAGCTAGAAATGTRGCCCACGCAGGGCCTTCAGTAGATTTAGCTATTTTCTCACTTCATTTAGCAGGAGCTTCATCTATTTTAGGGGCAATTAATTTCATTACAACTGTAATTAATATGCGATGATCCGGACTACGTCTAGAGCGTATCCCACTATTTGTATGGGCCGTRGTTATCACAGTAGTACTATTATTACTTTCCCTRCCTGTATTAGCAGGGGCTATCACTATACTACTAACTGAYCGTAATCTAAACACATCATTTTTTGATCCTGCTGGGGGCGGCGACCCCATTCTATATCAACACCTA

Consensus of 3 sequences of *Amynthas tontong* sp. n. **Paratype** CUMZ 3207, GenBank Accession No. HQ562073-HQ562076.

AACCCTATACTTCATTTTAGGAATTTGAGCTGGAATAATTGGAGCAGGAATAAGACTCCTTATTCGAATTGAGYTAAGACAGCCCGGATCATTCCTAGGAAGYGATCAACTATACAATACCATTGTTACAGCCCATGCATTCTTAATAATTTTCTTCCTRGTAATGCCAGTATTTATTGGGGGCTTTGGAAATTGATTACTTCCACTAATGTTGGGGGCCCCTGACATAGCTTTCCCACGACTAAATAATATGAGATTTTGACTACTTCCACCATCATTAATCCTTTTAGTTAGATCCGCRGCCGTTGAAAAAGGTGCGGGGACAGGATGAACTGTATACCCACCACTAGCAAGGAACATTGCCCATGCTGGCCCATCTGTAGATTTAGCAATTTTCTCACTACACTTGGCGGGGGCATCCTCAATCCTGGGGGCTATTAACTTCATTACCACAGTAATTAATATGCGATGATCTGGGCTGCGCTTAGAACGAATCCCCCTATTTGTATGAGCTGTAGTAATTACAGTAGTACTCCTACTACTATCTTTGCCCGTGCTGGCGGGAGCCATTACAATACTCTTAACAGATCGAAATCTTAATACATCATTCTTCGACCCTGCTGGCGGGGGCGACCCTATTCTATACCAGCACCTG
